# Biotic and Abiotic Stressors in Plant Metabolism

**DOI:** 10.3390/ijms25010121

**Published:** 2023-12-21

**Authors:** Laura Cornara, Manuela Mandrone, Antonella Smeriglio

**Affiliations:** 1 Department of Earth, Environment and Life Sciences, University of Genova, 16132 Genova, Italy; 2 Department of Pharmacy and Biotechnology, University of Bologna, Via Irnerio, 42, 40126 Bologna, Italy; manuela.mandrone2@unibo.it; 3 Department of Chemical, Biological, Pharmaceutical and Environmental Sciences, University of Messina, Viale Ferdinando Stagno d’Alcontres 31, 98166 Messina, Italy; antonella.smeriglio@unime.it

Plants are subject to a variety of biotic and abiotic stress that affect their metabolism, physiology, morphology, and growth. Nowadays, abiotic stress factors, high salinity, extreme temperatures, drought, mineral deficiencies, and soil contamination are all factors on which research has focused. Furthermore, adverse biotic factors, such as pathologies that are associated with microorganisms, together with attacks and interactions involving insects and herbivores and an increase in weed biomass, influence crop productivity, causing significant yield losses [1,2].

The mechanism of biotic and abiotic stress resistance in plants involves morphological, phytochemical, physiological, and molecular aspects. Among the various defensive strategies to tolerate adverse factors, there are alterations in anatomical features. For example, in many woody plants, root morphology and root system architecture can change to ensure plant survival under abiotic stresses [3]. In response to environmental stress, such as prolonged drought, plants can balance water uptake and transpiration by changing their leaf size, stomata density or cell wall thickness, waxy cuticle, cell density trichromes, etc. [4].

Moreover, a variation in the production of plant metabolites is also involved in the upregulation or downregulation of certain metabolic pathways to counteract stressful conditions. In this context, metabolomics is emerging as an interesting approach to evaluate the correlation between plant metabolites’ variations and environmental parameters, as demonstrated in the articles published in this Special Issue. In particular, the production of reactive oxygen species and metabolites, such as ascorbic acid, salicylic acid, jasmonic acid, terpenes, alkaloids, and polyphenols, trigger plant defense [5]. Another example is provided by the increased production of antioxidant compounds, especially flavonoids bearing catechol structures, to counteract the chilling stress in plant tissues due to a lowering in temperature during seasonal variation [6,7]. Water supply and soil features (i.e., clay content) are correlated to specific metabolomic variations in *Sorghum bicolor* L., affecting dhurrin (and related metabolites), amino acids, organic acids, and carbohydrate content in sorghum leaves. In addition, an increase in chlorogenic acid is registered as a consequence of predator attacks [8].

This Special Issue entitled “Biotic and Abiotic Stressors in Plant Metabolism” of the *International Journal of Molecular Sciences* includes a total of seven original articles providing new information about the most important factors involved in plant tolerance to environmental stress. 

Cornara et al. [9] investigated the influence of pedoclimatic characteristics on the rare species *Saponaria sicula* Raf. which grows exclusively on the limestone cliffs of the Madonie and on the volcanic sands of Etna. A comparison between the plants grown in these two different sites highlighted that pedoclimatic conditions influenced the morpho-anatomical characteristics of these leaves. The leaves from Madonie, in fact, had a greater quantity of calcium oxalate druses in the mesophyll and a greater content of polyphenols, also confirmed by the different phytochemical profiles of these two extracts. These results were also in agreement with the biological activity tested, which indicated that the leaf extract obtained from plants collected in Madonie had the strongest antioxidant and anti-inflammatory activity.

Specific metabolites, in particular alkaloids and isoflavones, are associated with plant defense from pathogens [10]. To study the potential mechanisms of resistance and susceptibility to the pathogen fungi *Fusarium graminearum* (FHB), Dong et al. [11] inoculated FHB in three different varieties of *Triticum aestivum* L. Through untargeted metabolomics, numerous metabolites (both secondary and primary) were found to be upregulated as a consequence of the infection. In particular, the antifungal metabolites phenylalanine, spermidine, tryptophan, malic acid, fumarate, fructose, nicotinamide, and cinnamic acid were upregulated in the three varieties, and benzoic acid, glucosamine, and kaempferol-3-*O*-glucoside were upregulated only in the highly resistant variety. Among the metabolites involved in plant immunity, agmatine, succinate, and jasmonic acid were upregulated in the three varieties, and proline, argininosuccinate, gamma-aminobutyrate, m-salicylic acid, and guanosine were upregulated only in the highly resistant variety. 

Testing a selection of these compounds in a fungistatic experiment, phenylalanine and malic acid exhibited a significant inhibiting effect on *F. graminearum* growth. 

Abiotic stress is also an important factor that induces metabolomic change in plants. Yu et al. [12] explored the variation in the metabolome as a consequence of drought stress in *Elymus sibiricus* drought-tolerant and drought-sensitive genotypes. This study led to identifying potential biomarkers of drought stress in *E*. *sibiricus* different genotypes. Moreover, it also contributed to a better understanding of the mechanisms of drought resistance in this plant. In particular, by simulating long-term and short-term drought stress, some common metabolites were found to be upregulated in both genotypes. Ten metabolites, including 3-amino-2-methylpropanoic acid, coniferin, R-aminobutyrate, and others, showed differential accumulation patterns under short-term drought stress. There were twelve metabolites, among which L-proline, L-histidine, N-acetylglycine, and others were associated with drought stress. 

Interestingly, in both genotypes, no significant accumulation of flavanones, pyridines, and phenol amides was found under drought stress in *E. sibiricus*.

3-amino-2-methylpropanoic acid, coniferin, R-aminobutyrate, galactinol, D-glutamic acid, vitexin, and trigonelline were accumulated preferentially in the drought-tolerant genotype under short-term drought, while L-glutamic acid, L-arginine and L-ornithine were accumulated more in the drought-sensitive genotype under short-term drought. Similarly, L-proline, L-histidine, N-acetylglycine and betaine were accumulated in the drought-tolerant genotype and N2-acetyl-L-lysine, 3-ureidopropionate, vitamin U, 1,2-benzenedicarboxylic acid, IAA-Asp, 1-methylpiperidine-2-carboxylic acid, L-proline, and D-proline betaine accumulated in the drought-sensitive genotype. 

Metabolomics, together with other “omics” techniques, resulted in great interest in implementing genetic transformation strategies on genetically modified plants. For instance, Zhang et al. [13] studied the regulation patterns of gene expression and the metabolite contents during leaf wilting in tobacco leaves caused by the transfection of the morphogenic transcription factor *ZmWus2*, which is widely used to promote plant transformation efficiency. Both transcriptomic and untargeted metabolomic analyses were performed on tobacco leaves in their healthy and wilted states after *ZmWus2* transient overexpression. *ZmWus2* transformation resulted in the activation of inositol trisphosphate and glycerol-3-phosphate metabolism while also upregulating plant hormone signaling and downregulating photosystem and protein folding pathways. Phenylpropanoid compounds and various lipid classes, including steroid synthesis, were found to be increased under this condition. In addition, transcription factors such as ethylene-responsive factors, the basic helix–loop–helix factors, and MYBs were found to be regulated by *ZmWus2*.

In response to abiotic stress, secondary plant metabolites exert an allelopathic effect, offering valid alternatives to chemical herbicides that are not only harmful to humans, animals, and the environment but, over time, lose effectiveness, quickly causing the onset of resistance phenomena. 

Research is currently focusing on the search for new natural agents, such as ailanthone, a quassinoid naturally produced by *Ailanthus altissima* (Mill.) Swingle, which suppresses the growth and germination of other plants. Recently, Hopson et al. [14] investigated the effect of this secondary metabolite on three *Arabidopsis thaliana* ecotypes through a transcriptome and physiology analysis. From a physiological point of view, they observed a decrease in root growth as well as a high proline and reactive-oxygen-species (ROS) accumulation in response to ailanthone stress. Furthermore, the transcriptome analysis revealed differentially expressed genes, highlighting that ailanthone induces the significant alteration of several stress, development, and hormone metabolism pathways. Another recent study by Chen et al. [15] evaluated the response of *Zea mays* L. to cold stress via sequence and promoter analysis on five genes previously identified by the same authors using transcriptome sequencing analysis and codifying for a key protein involved in plant abiotic stress, the dehydrin. The results showed that the ZmDHN15 gene was significantly upregulated. To verify the role of this gene in cold stress, the authors overexpressed it in yeast and *Arabidopsis*, finding that the expression of this gene can significantly improve the cold resistance of yeast. Furthermore, under cold stress, ZmDHN15-overexpressing *Arabidopsis* showed a lower oxidative marker content and electrolyte leakage in comparison with wild-type plants, as well as a higher seed germination rate, seedling survival rate, and chlorophyll content. These results provided useful starting points for the improvement of genetic crops and germplasm innovation.

On the contrary, regarding plants’ response to biotic stress, Sun et al. [16] investigated the effect of the labdane diterpenoid cis-abienol, abundantly produced by glandular trichomes of *Nicotiana* spp., against the bacterial wilt of tomatoes via pot-inoculation experiments. The treatment of the roots with 60 µg/mL of cis-abienol for 2-3 consecutive applications with 3–6 day intervals showed the most effective results. The mode of action involved an increase in the antioxidant enzyme activity and the induction of the defensive signal transduction, leading to the upregulation of several genes involved in the mitogen-activated protein kinase cascade and jasmonate ZIM-domain. This determines, accordingly, an increase in jasmonic and salicylic acid synthesis, which, in turn, control the expression of flavonoid biosynthetic genes and the content of phytoalexins in tomato roots improving the bacterial wilt resistance of tomato plants.
